# Locoregional Therapies for Hepatocellular Carcinoma with Portal Vein Tumor Thrombus

**DOI:** 10.1007/s12029-025-01280-2

**Published:** 2025-07-23

**Authors:** Ramanpreet Singh, Mina S. Makary

**Affiliations:** 1 Northeast Ohio Medical University, Rootstown, OH 44272 USA; 2 Division of Vascular and Interventional Radiology, Department of Radiology, The Ohio State University Medical Center, Columbus, OH 43210 USA

**Keywords:** Hepatocellular carcinoma, Portal vein tumor thrombus, Transarterial Chemoembolization (TACE), Transarterial Radioembolization (TARE), Hepatic Artery Infusion Chemotherapy (HAIC), Stereotactic Body Radiotherapy (SBRT)

## Abstract

Portal vein tumor thrombus (PVTT) develops in up to half of patients with hepatocellular carcinoma (HCC) and historically signifies advanced-stage disease with limited treatment options and poor prognosis. Systemic therapy has been the standard treatment for HCC with PVTT, but this review highlights the potential of image-guided locoregional therapies including transarterial chemoembolization (TACE), transarterial embolization (TAE) radioembolization (TARE), hepatic arterial infusion chemotherapy (HAIC), and ablative or radiotherapeutic approaches to improve outcomes in this challenging context. We will summarize current evidence and clinical experience demonstrating that these modalities can achieve meaningful tumor control and extend survival, especially when tailored to tumor burden and PVTT extent or combined with systemic treatments. These findings underscore that aggressive locoregional treatment can be a valuable component of multidisciplinary management for advanced HCC, offering select patients an improved prognosis despite PVTT.

## Introduction

**Fig. 1:**
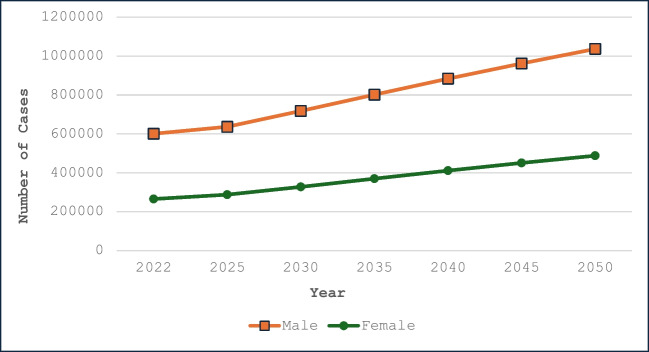
Projected new liver cancer cases in males and females globally from 2022 to 2050. Data sourced from GLOBOCAN 2022, https://gco.iarc.who.int/tomorrow/en/dataviz/trends?types=0&cancers=11&years=2050&multiple_populations=1&group_cancers=0&multiple_cancers=0&num_path=4&sort_by=value1&single_unit=50000 Created in Microsoft Excel

HCC is a significant health burden worldwide and it is the third leading cause of cancer-related deaths with the highest incidence of liver cancers observed in Mongolia and China [1, 2]. HCC is biologically and morphologically diverse and may present as a unifocal or multifocal mass, or as diffuse malignant infiltration of the liver [3]. Consistent improvements have been seen in screening and surveillance, yet the incidence and mortality from HCC are rising at an alarming rate, and it does not seem to be slowing down [4]. By 2050, the global deaths from liver cancer are expected to exceed 900,000 for men and 500,000 for women annually, as shown in Fig. 1, with a 5-year survival rate of less than 20% [2]. This tremendous rise in burden is concerning and emphasizes an urgent need to optimize treatment strategies, especially for patients with advanced disease complicated with PVTT, where the prognosis is bleak despite advances in systemic therapy.

PVTT is present in 10–40% of individuals diagnosed with HCC. The development of PVTT signifies an advanced disease stage (Barcelona Clinic Liver Cancer (BCLC) Stage C), leading to portal hypertension and rapid tumor progression, and intrahepatic spread [5]. The prognosis of HCC with PVTT is poor and without intervention the overall survival (OS) rate is 2–4 months [2, 6]. This leads to a dark and grim outlook, where PVTT urgently needs innovation in therapeutics.

Minimally invasive image-guided locoregional therapies show promise in treating HCC complicated by PVTT. The therapeutic modalities of interest are transarterial chemoembolization (TACE) and bland arterial embolization (TAE), transarterial radioembolization (TARE) with Yttrium-90 (internal radiation therapy), hepatic artery infusion chemotherapy (HAIC), percutaneous tumor ablation (radiofrequency (RFA), microwave ablation (MWA), or cryoablation (CRA)), and high-precision radiotherapies. These therapies have shown to improve outcomes in HCC with PVTT in multiple clinical trials and stand to challenge systemic therapy as the only option for treatment. Throughout this article, the term TACE refers specifically to conventional transarterial chemoembolization (cTACE) unless otherwise specified. When discussing drug-eluting bead TACE, the term DEB-TACE is used explicitly to distinguish it from cTACE. The goal of this review is to evaluate the role of these image-guided locoregional therapies by being the front-line interventions or as adjuncts to other therapies in the management of HCC with PVTT.

### Cheng’s Classification of PVTT (Types I-IV)

**Fig. 2:**
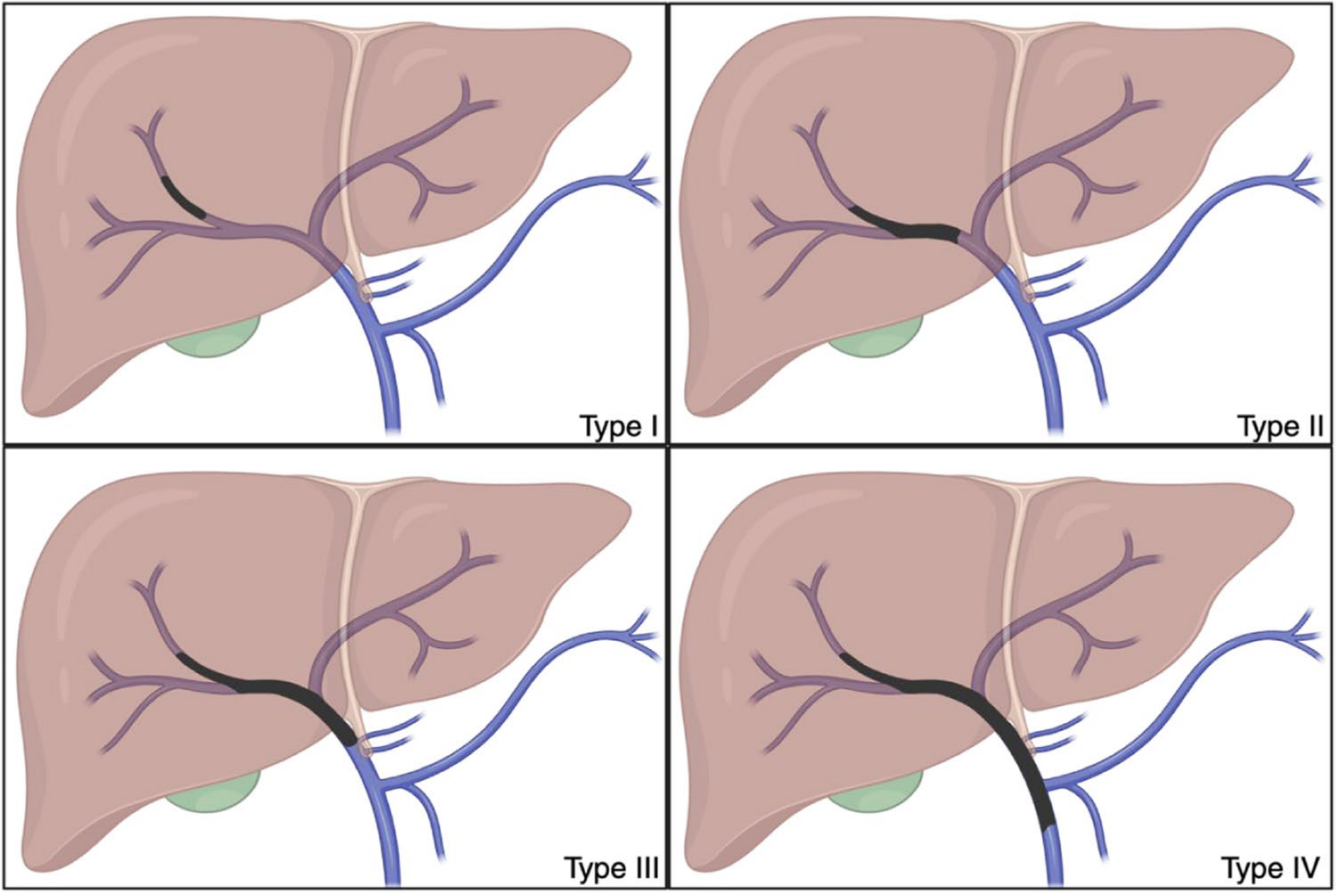
Cheng Classification of PVTT in HCC. Top left (Type I): Tumor thrombus in segmental branches. Top right (Type II): Thrombus in right or left portal vein (lobar branch). Bottom left (Type III): Thrombus in main portal vein trunk. Bottom right (Type IV): Thrombus extending from the main portal vein into the superior mesenteric vein (SMV). Created in BioRender. Singh, R. (2025) https://BioRender.com/qtc877t -Thrombus

Having a foundational understanding of the various classification systems is essential in defining the extent of portal vein thrombosis. The Cheng classification is an anatomically focused four-tier system that categorizes PVTT by its extent within the portal venous system [7]. Type I PVTT involves segmental branches, Type II PVTT extends into first-order branches, Type III PVTT occupies the main trunk, and Type IV PVTT is the most advanced extending into the superior mesenteric vein [7, 8]. Clinically, PVTT classifications usually refer to the radiologically visible thromboses Types I–IV as shown in Fig. 2. The Cheng classification system helps stratify HCC patients by risk and helps guide therapy as it correlates with survival and informs treatment selection.

### Japanese Vp and Xu Classification Systems

Another classification system that parallels Cheng’s system was developed by the Liver Cancer Study Group of Japan, the Vp classification system. This system defines Vp0 as no portal vein involvement, Vp1 indicating tumor thrombus distal to second-order branches, Vp2 involves second-order branches (segmental), Vp3 indicates thrombus in a first-order branch (right or left portal vein), and Vp4 indicates tumor thrombus in the main portal vein trunk [7]. The Japanese classification system is used widely in East Asia and has been shown to predict prognosis incrementally with higher Vp stage [9].

There are simpler binary classifications put forth by other groups, such as the Xu classification. This system classifies PVTT into Type A and Type B. Type A indicates involvement of the main portal vein or both first-order branches, whereas Type B is confined to one first-order branch (left or right) [7]. Of note, Cheng’s Type I vs II roughly correspond to Xu’s Type B (since Types I/II are limited to one side), while Cheng’s Types III/IV correspond to Xu’s more extensive Type A category.

### The Child–Pugh and BCLC Staging Systems

The Child–Turcotte–Pugh (CTP or Child–Pugh (CP)) shows higher points reflecting worse hepatic reserve. Total scores of 5–6 define class A (well-compensated liver), 7–9 class B (significant impairment), and 10–15 class C (decompensated) [10]. The BCLC stages are classified as very early/early (Stage 0/A), intermediate (B), advanced (C), or end‐stage (D). Stage 0/A has a single or few small tumors, preserved liver function (CP-A), and Eastern Cooperative Oncology Group (ECOG) PS 0, curative therapies (hepatic resection, liver transplantation, or image-guided ablation) are recommended [11]. Stage B (intermediate) encompasses multinodular HCC, asymptomatic, with preserved function. These patients are directed to transarterial therapies (typically TACE) [11]. Stage C (advanced) has vascular invasion or extrahepatic spread, or symptomatic tumors (PS 1–2). The BCLC recommends systemic therapy (sorafenib/Lenvatinib etc.). Stage D (terminal) is decompensated liver disease (Child–Pugh C) and/or poor PS (3–4). Only best supportive care is advised [11].

Overall, all these classification systems share the central principle that more proximal PVTT equates to more advanced disease and worse prognosis [7, 12].

## Locoreginoal Therapies for HCC with PVTT

### Overview and Historical Context of TAE and TACE

**Fig. 3:**
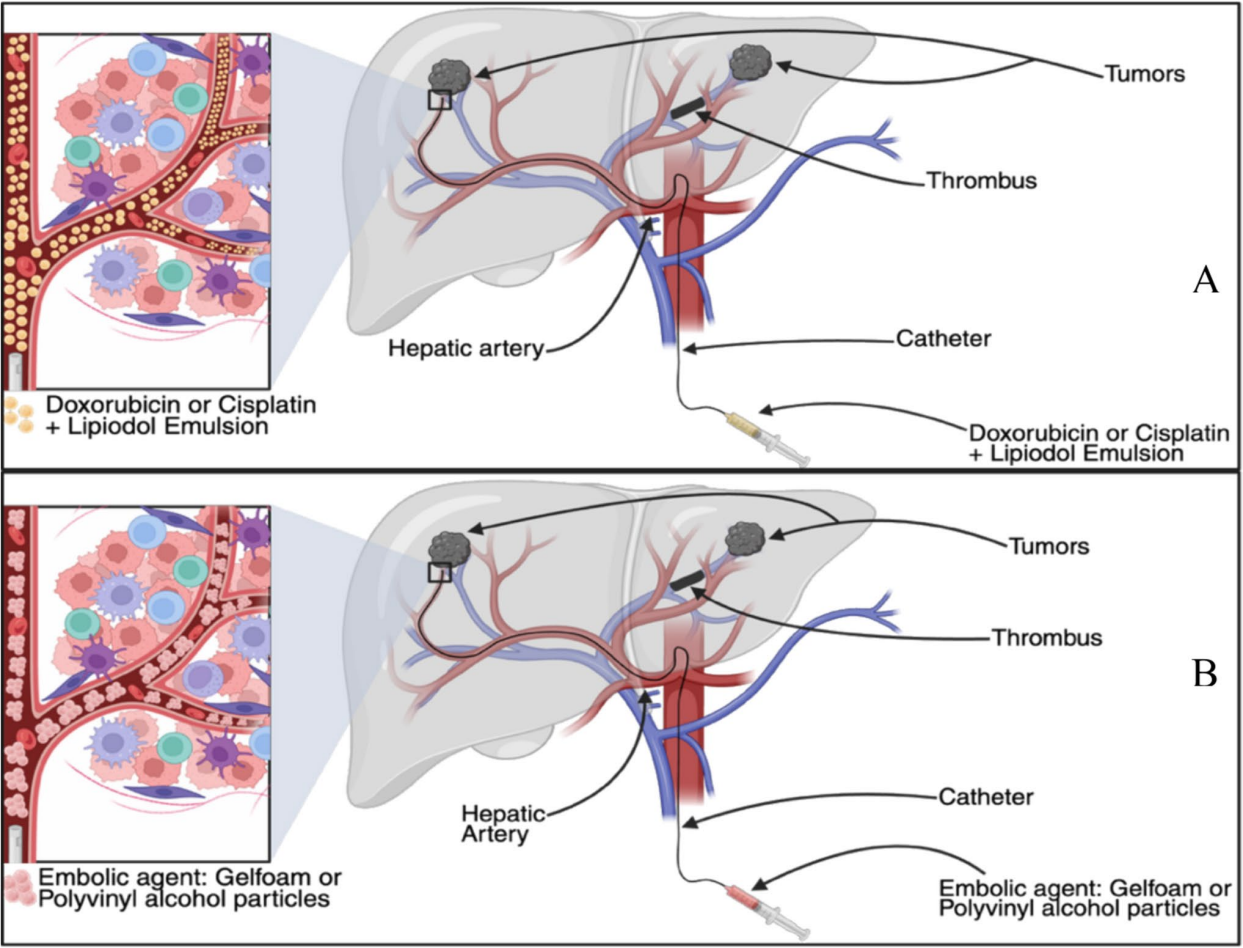
Selective Mechanism of cTACE of HCC in PVTT. A microcatheter is advanced into a tumor-feeding branch of the hepatic artery allowing for targeted intra-arterial delivery of doxorubicin or cisplatin mixed lipiodol. Part A shows the chemotherapeutic agent emulsified with lipiodol and injected into the tumor’s arterial supply. Part B shows the next step of administering embolic agents such as gelfoam or PVA particles. Created in BioRender. Singh, R. (2025) https://BioRender.com/phjby1z

TAE and TACE for HCC originated in the late 1970 s and early 1980s. TAE is the injection of embolic particles, into the hepatic artery without chemotherapeutic drugs, and TAE was used advantageously due to the fact that HCC derives the majority of its blood supply from the hepatic artery [13]. By occluding arteries that feed tumors with particles (initially polyvinyl alcohol (PVA), gelatin sponge, coils, later calibrated microspheres), ischemic tumor necrosis can be induced with relatively limited effect on healthy parts of the liver. Over time, refinements in catheter technology and imaging guidance (digital subtraction angiography (DSA) and cone-beam computed tomography (CBCT)) have enabled more precise embolization of arteries that feed tumors. This was further built on by combining arterial infusion of chemotherapy with embolization, and thus TACE was developed, as shown in Fig. 3, in the early 1980 s [14]. The physiologic rationale was compelling because 90–100% of HCC lesions derive their blood supply from the hepatic artery, whereas normal liver parenchyma is mostly fed by the portal vein [15, 16]. Risks included post-embolization syndrome characterized by abdominal pain, nausea, vomiting, fevers, and chills. This was seen in almost all patients, and in patients with poor liver function or portal vein thrombosis that could have hepatic infarction and liver failure occur [3, 17, 18]. Two landmark randomized trials provided high-level evidence that TACE conferred a survival advantage in unresectable HCC [14, 19]. They also highlighted safety limitations; in Lo’s study, patients treated with TACE had a higher incidence of liver failure deaths, highlighting the need for careful patient selection [19].

Over the decades there has been a significant evolution in TACE and TAE, and patient selection criteria. By the 2000 s, DEB-TACE was developed, introducing microspheres that slowly release chemotherapy into the tumor bed [20]. This innovation aimed to improve local drug delivery and reduce systemic toxicity. Studies have shown that DEB-TACE is non-inferior to cTACE in efficacy, with comparable tumor response and survival rates [21]. In one study, 471 patients with locally advanced HCC (LA-HCC) underwent DEB-TACE. Results showed complete radiologic response in 120 (25.5%) patients, comparable to 28% for cTACE [22]. Some studies suggest DEB-TACE may confer lower rates of systemic side effects and liver toxicity due to the controlled drug release [21, 23]. Overall outcomes for DEB-TACE and cTACE have consistently proven superior to conservative therapy [7, 16].

### TAE and TACE in HCC with PVTT

TACE has increasingly been applied to HCC patients with PVTT, challenging the conventional principle that PVTT precludes arterial intervention. The phase III LAUNCH trial investigated cTACE combined with systemic Tyrosine kinase inhibitor (TKI) Lenvatinib in advanced HCC (a significant portion of patients having PVTT) [24]. The combination of TACE + Lenvatinib achieved a median OS of 17.8 months, significantly longer than the 11.5 months with Lenvatinib alone [24]. Another study focusing specifically on HCC with PVTT had 265 PVTT patients; those who received immunotherapy plus TACE had a median OS of 19.0 months versus 13.0 months with immunotherapy alone (*p* < 0.001) [25]. These findings align with the general trend that multimodal therapy yields the best outcomes in HCC with vascular invasion. Taken together, these trials and series consistently show median survivals greater than 12 months for PVTT patients receiving TACE in combination with systemic or regional adjunct therapies [26, 27], whereas median survival rarely exceeds 6–8 months with systemic therapy alone in similar patient populations [28]. This represents a substantial improvement over historical outcomes and underscores the increasing role of TACE-based combination regimens in advanced HCC. Additionally, modern interventional radiology employs CBCT during angiography to detect small tumors and delineate feeding vessels that might be missed on planar DSA imaging. The use of CBCT with automated feeder detection software showed significant improvement in treatment response rates and prolonged survival by enabling super-selective targeting of tumor arteries [29].

TACE therapy to the side ipsilateral to tumor and thrombus is generally not recommended; however, a recent study suggests that TACE can be safely performed in patients with ipsilateral PVTT. The study required that the PVTT be ≥ 1 cm from the main portal vein and limited to Vp1–Vp3 disease, with preserved liver function [30]. In contrast to cTACE, DEB-TACE is generally not recommended in patients with ipsilateral PVTT. The prolonged embolic effect of drug-eluting beads can lead to sustained ischemia, increasing the risk of liver infarction in regions already affected by PVTT. These findings highlight that in selected patients, lobar or segmental TACE ipsilateral to PVTT may offer substantial benefit without causing hepatic decompensation [30].

Combination therapy strategies have emerged to maximize efficacy in HCC with PVTT while mitigating the limitations of TACE alone. A multicenter retrospective study treated HCC with main-branch PVTT using combined TACE + Lenvatinib + PD-1 inhibitors. Over a median 18.0 months follow-up, the ORR in the liver was 68.3%, the median PFS was 14.5 months, and median OS was 21.7 months [31]. This triple therapy was well tolerated, suggesting that aggressive loco-regional plus systemic therapy can yield ORR ~ 70%, long PFS (~ 14 months) and OS ~ 22 months even in main-branch PVTT [31]. Outcome data are increasingly stratified by the extent of PVTT since this factor profoundly influences prognosis after TACE. Generally, patients with thrombus confined to segmental or lobar branches of the portal vein fare much better than those with main portal trunk involvement. In practice, many centers will still attempt locoregional treatment for lobar PVTT but refrain from TACE when the portal trunk is fully occluded. TACE was once avoided in HCC with PVTT, but now it has shown evolving safety and efficacy with newer techniques and careful patient selection.

### Overview and Historical Context of TARE

**Fig. 4:**
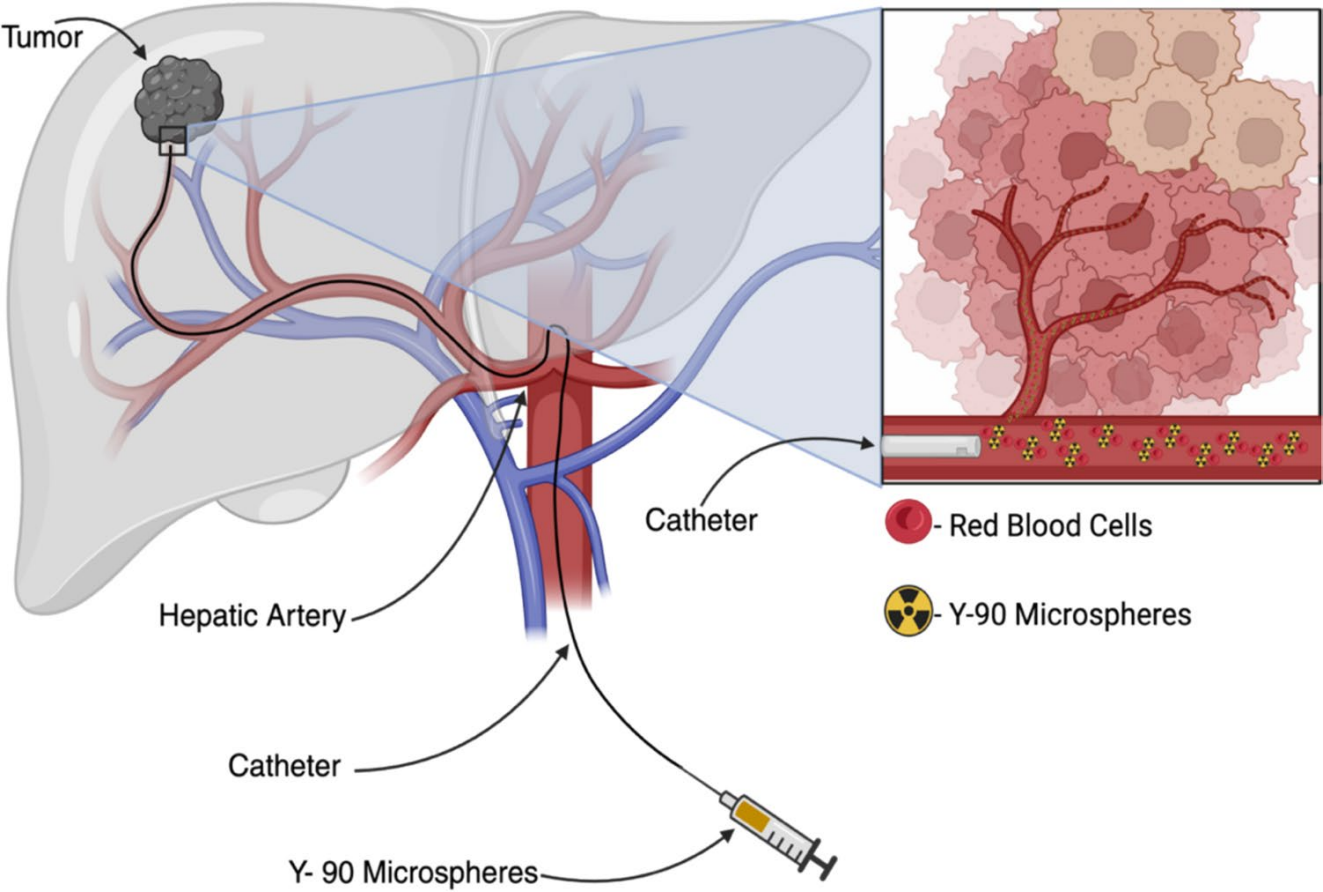
Mechanism of TARE for HCC. TARE, also known as selective internal radiation therapy (SIRT), for the treatment of HCC. A microcatheter is inserted into the hepatic artery and advanced to the tumor-feeding branch. Next, yttrium-90 microspheres are infused, lodging in the tumor’s microvasculature. This delivers a high dose of internal radiation while sparing adjacent healthy liver tissue Created in BioRender. Singh, R. (2025) https://BioRender.com/cc8m5qf

TARE using yttrium-90 microspheres, as shown in Fig. 4, was first conceived in the 1960 s and adopted clinically in the early 2000s. TARE delivers high-dose β-radiation to the tumor while preserving non-tumoral liver perfusion. Two microsphere platforms are dominant. The resin microspheres (SIR-Spheres) have ^90^Y absorbed onto the surface and glass microspheres (20–30 µm) have ^90^Y embedded in a glass matrix [32]. Glass spheres have higher specific activity per sphere, permitting smaller numbers of spheres and very high tumor doses per particle, while resin spheres use many more particles with lower activity each. Over the past decade, evidence has accumulated that personalized dosimetry yields better outcomes [33].

Recent prospective studies and trials have clarified TARE outcomes in HCC with PVTT. In the prospective international Carbon Ion Radiotherapy (CIRT) cohort (422 unselected HCC patients), 75 patients were BCLC C (with PVTT). On multivariable analysis, the presence of any PVTT was associated with worse survival; however, optimized dosimetry showed markedly improved outcomes. This prospective series compared standard BSA dosing to partition dosimetry, and patients planned with partition modeling had a median OS of 23.4 months vs only 13.4 months for traditional BSA-based dosing (*p* < 0.0001) [34]. In other words, TARE outcomes in advanced HCC, even with PVTT, depend critically on achieving high tumor doses.

A landmark randomized trial (DOSISPHERE-01) prospectively evaluated personalized ^90^Y glass TARE in locally advanced unresectable HCC (often with PVTT) using either standard (120 Gy) or boosted dosimetry. In that phase-II RCT, the subgroup of patients with macrovascular invasion showed a median OS of 22.0 months (95%CI 10.3–36.5) in the personalized-boost arm vs only 9.5 months (5.3–17.6) in the standard arm [35]. Further analysis of DOSISPHERE confirmed a dose–response relationship where patients receiving a planned tumor dose (PD) ≥ 150 Gy had median OS 22.9 months vs only 10.3 months if PD < 150 Gy [35]. These contemporary data underscore that with personalized TARE, even PVTT-bearing tumors can achieve long remissions, provided a sufficiently high dose is delivered.

### TARE in HCC with PVTT

Modern series report substantially better outcomes with TARE, especially when PVTT is limited to segmental or lobar branches. A 2023 multicenter study found that in matched cohorts with segmental/lobar PVTT, ^90^Y TARE yielded a median OS of 28.2 months, compared to only 7.2 months in patients treated with sorafenib or Lenvatinib [36]. ORR (mRECIST) exceeded 50% with TARE (vs ~ 12–15% with TKIs) [36]. Even in more advanced cases, TARE yields meaningful control. A single-center Indian study reported median OS ≈15 months and ORR 58% after ^90^Y in advanced HCC including PVTT [37]. A 2024 phase-II pilot trial treated 27 poor-prognosis HCC patients (39% had PVTT) with sequential ^90^Y radioembolization followed by pembrolizumab. The combination was safe and yielded encouraging activity and median PFS was 9.95 months, and median OS was 27.3 months with an overall response rate of 30.8% [38]. These impressive outcomes suggest that even PVTT-HCC can achieve durable disease control by integrating TARE with immune checkpoint blockade.

In summary, contemporary prospective data support TARE as a potent, liver-directed therapy for HCC with portal vein invasion. TARE applied through personalized dosimetry can achieve substantial survival (often > 20 months) and tumor control in selected PVTT patients [35, 39]. Outcomes are best in those with limited PVTT and preserved liver function, and main-branch PVTT confers much poorer prognosis [34]. In these indications, TARE is now a well-established component of the advanced HCC treatment algorithm, used either alone or combined with systemic/adjunct therapies, with ORR on the order of 50–60% [36, 37] while other patients continue to rely on systemic therapy as the mainstay.

### Overview and Historical Context of HAIC

HAIC was developed as a locoregional strategy for unresectable HCC. In patients with PVTT, TAE/TACE risks ischemia, whereas continuous infusion via the hepatic artery can deliver high drug concentrations directly to both tumor and thrombus while sparing normal liver parenchyma [40]. In one large propensity-score–matched cohort, median survival was 16 vs 6 months in PVTT patients [41]. Subsequent prospective trials formally tested HAIC against sorafenib and in a randomized Korean study of 58 PVTT patients, HAIC significantly outperformed sorafenib (median OS 14.9 vs 7.2 months; *p* = 0.012), with longer time to progression (TTP) (4.4 vs 2.7 months; *p* = 0.010) and higher disease control rate (DCR) [42]. These pioneering studies established HAIC as at least comparable to sorafenib and justified further development. In parallel, a landmark phase III trial showed FOLFOX-HAIC dramatically improved survival vs TACE (median OS 23.1 vs 16.1 months), illustrating the potency of modern HAIC regimens in advanced liver cancer [43].

Over time, HAIC techniques evolved to use oxaliplatin-based FOLFOX regimens. In general, evolving HAIC practice has focused on maximizing local drug exposure (via continuous infusion pumps and pressurized infusion) and combining agents (leucovorin (LV) to modulate 5FU) to optimize response while managing toxicity.

### HAIC in HCC with PVTT

In the last three years, multiple prospective and observational studies have quantified HAIC outcomes in PVTT. In a Phase II randomized trial, 64 HCC patients with PVTT received either sorafenib alone or sorafenib plus HAIC. The sorafenib + HAIC arm had markedly superior survival with median OS of 16.3 vs 6.5 months (HR 0.28; *p* < 0.001), and median PFS 9.0 vs 2.5 months (HR 0.26; *p* < 0.001) [44]. ORR (RECIST) was 41% with HAIC combination vs only 3% with sorafenib (*p* < 0.001) [44]. This trial also documented acceptable toxicity (grade 3–4 diarrhea 22% vs 16%, thrombocytopenia 22% vs 0% with and without HAIC) [44]. In sum, adding HAIC tripled median survival in this high-risk cohort. These findings echo earlier trials and reinforce that multi-drug hepatic infusion can dramatically improve local control and OS in PVTT.

Similarly, combining HAIC with anti-angiogenic TKIs and immunotherapy has shown high activity with one study observing an ORR of 74.1%, and even higher PVTT response (best 98.1%) [45]. Median OS was 10.0 months and PFS 5.0 months [45]. Other retrospective studies that included patients with PVTT confirm similar efficacy where the ORR was 52.7% (RECIST) and median OS 14.3 months, again underscoring the synergy of HAIC with targeted immunotherapy [46]. Another study compared 35 patients receiving HAIC plus Lenvatinib + tislelizumab (triple therapy) vs 120 receiving HAIC alone, all with Vp4 PVTT. After propensity matching, median OS was 23.2 vs 6.9 months (HR 0.333; *p* < 0.001), and median PFS 6.6 vs 2.4 months (HR 0.403; *p* = 0.002) [47]. This demonstrates a profound survival benefit by adding systemic immunotherapy/targeted agents to HAIC in end-stage PVTT.

In summary, HAIC is a highly active locoregional therapy for HCC with PVTT. Current research focuses on integrating HAIC into multimodal regimens and early evidence suggests that HAIC plus targeted immunotherapy can substantially extend survival in advanced PVTT [40, 47]. Patient selection remains critical and optimal candidates have adequate hepatic reserve, limited extrahepatic disease, and large intrahepatic tumors with PVTT. As systemic therapies advance, HAIC is likely to remain an important option and merits further study in global clinical trials.

### Overview and Historical context of Ablation

**Fig. 5:**
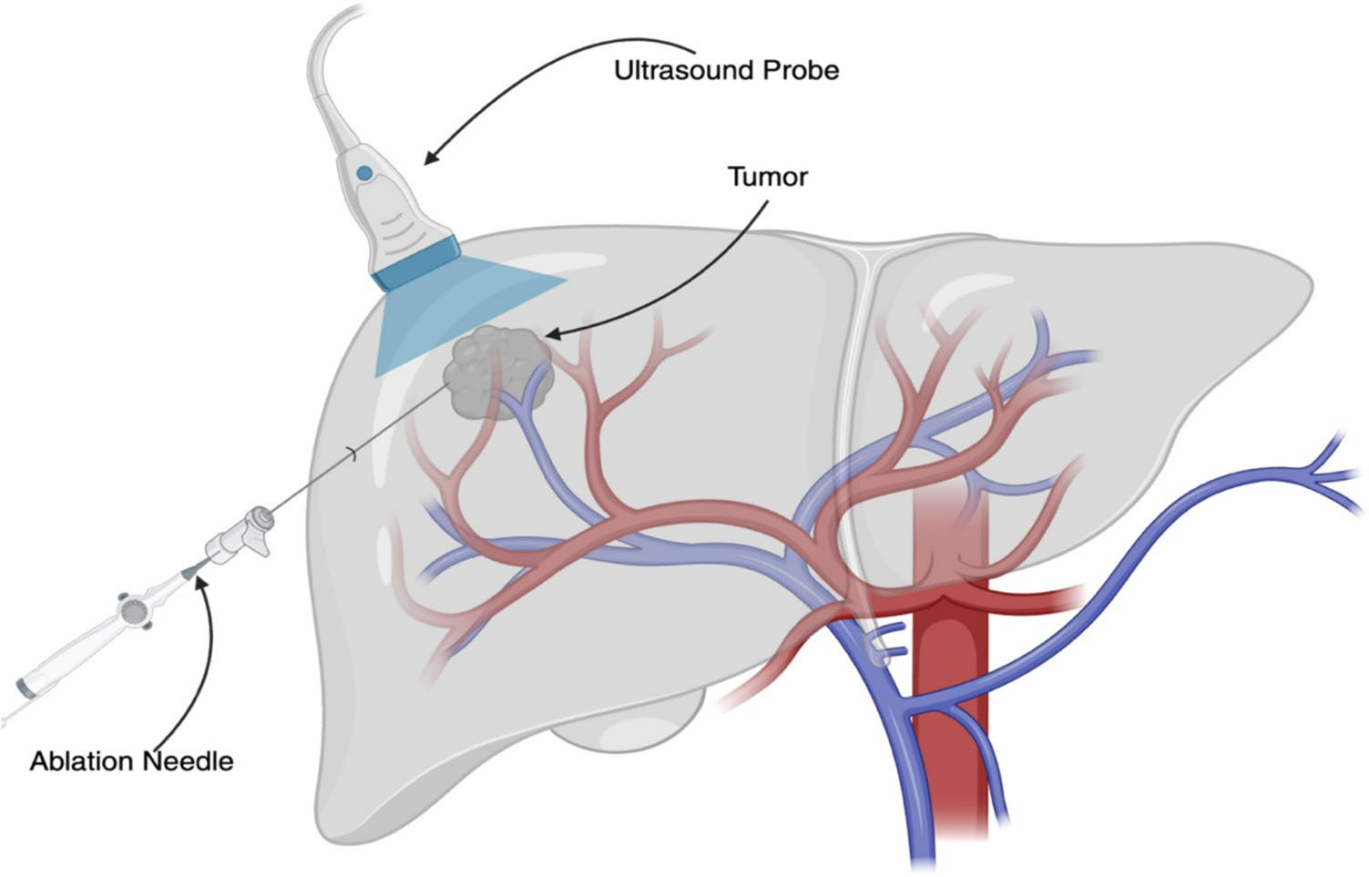
Ultrasound-guided percutaneous tumor ablation for HCC. A percutaneous image-guided thermal ablation for HCC. The tumor is visualized using real-time ultrasound imaging facilitating accurate placement of the ablation probe Created in BioRender. Singh, R. (2025) https://BioRender.com/ne581yd

RFA, MWA, and CRA were introduced in the 1960s–2000s as image‐guided methods to achieve local tumor eradication in HCC. RFA uses alternating current to generate heat, while MWA uses electromagnetic waves; both induce coagulative necrosis of tumor tissue. CRA destroys tissue by rapid freezing to − 20 °C or below, causing ice crystal formation and ischemic injury. Early RFA procedures relied on ultrasound (US) guidance, as shown in Fig. 5, which can struggle to visualize PVTT. Modern practice often uses real-time fusion imaging that overlays prior CT or magnetic resonance imaging (MRI) onto live ultrasound, greatly improving lesion detection.

In practice, ablation of PVTT is often combined with other therapies. Common combinations include TACE plus ablation or ablation as an adjunct to resection or transplantation after downstaging. Collectively, these technical advances and combinations reflect a trend toward multi-modality management of HCC + PVTT, leveraging ablation as one component of therapy.

### Ablation in HCC with PVTT

Recent studies generally confirm that ablation can be done safely in select patients, often yielding median survivals measured in many months. A prospective dual-center study of 60 patients treated with endovascular RFA and portal stenting (percutaneous portal vein recanalization-endovascular placement of radiofrequency ablation-stenting (PVR-EPRFA-ST)) reported technical success in 90% of cases [48]. In that series, 68.5% of patients had improved portal hypertension or liver function, and portal vein patency was maintained for a median of 13.4 months [48]. In retrospective cohorts, ablative treatment of PVTT also showed encouraging results. A large single-center Chinese series compared 120 patients receiving PVR-EPRFA (with subsequent therapies) to 96 patients receiving TACE alone [49]. The RFA group had a much higher PVTT response and longer median OS (15.7 vs 11.3 months) [49]. Though the OS difference did not reach statistical significance, this suggests a trend toward benefit from direct PVTT ablation. In summary, modern RFA cohorts report a better median OS, limited PVTT, good liver function, and low complication rates.

MWA was particularly attractive for tumors near major vessels, a common scenario in HCC with PVTT. Over the past two decades, MWA technology has evolved from low-power, single-probe systems to advanced antenna arrays capable of ablating 3–5 cm tumors in a single session. One study found that MWA exhibited better local tumor control than RFA, particularly for tumors adjacent to the portal vein [50]. Clinically, the ability to create larger ablative margins has also enabled MWA to tackle multinodular or larger tumors often present with PVTT. Recent studies have solidified MWA’s role in advanced HCC with PVTT, mostly in combination with other therapies. In one study, TACE + MWA was used for PVTT in patients intolerant to sorafenib/Lenvatinib. Median OS was ~ 17 months with TACE–MWA, comparable to ~ 13.5 months in a TACE plus TKI cohort (no statistical difference) [51]. These data suggest MWA-based locoregional therapy can approximate the survival achieved with targeted agents in select PVTT cases, offering a viable option when systemic therapy is contraindicated. Another study reported a landmark propensity-score study of 87 advanced HCC patients (most with PVTT) receiving synchronous TACE + MWA with or without anti–PD-1 checkpoint inhibitor therapy [52]. The triple modality group (TACE + MWA + PD-1 inhibitor) achieved an objective response in 57% of intrahepatic tumors and significantly prolonged survival relative to the dual therapy group (TACE + MWA alone). Median OS improved to 17.0 months with immunotherapy vs 8.5 months without (*p* < 0.001) [52]. These findings highlight that adding immunotherapy to locoregional treatment can synergistically improve outcomes in PVTT, presumably by eradicating microscopic metastases and bolstering anti-tumor immunity after MWA-induced antigen release. MWA was historically introduced as a faster thermal ablative method. It has seen significant technological refinement and clinical adoption. Modern studies demonstrate that MWA combined with TACE improves survival compared to TACE alone and the addition of systemic/immune therapies can further augment outcomes [53, 54].

A key advantage in HCC/PVTT is that CRA is less affected by blood flow because cold is not dissipated as quickly as heat, so the lethal ice ball can envelop tumors bordering the portal vein. Additionally, the frozen ice ball is clearly visible on CT/ultrasound, allowing precise monitoring of ablation margins in real time [55, 56]. Modern cryoprobes can be deployed percutaneously in multiples, enabling treatment of tumors > 5 cm by overlapping freeze zones.

One multicenter randomized controlled trial compared percutaneous CRA vs RFA in early-stage HCC. While this trial enrolled mostly small tumors (≤ 3–4 cm) without PVTT, its findings are relevant to technique efficacy. CRA achieved significantly lower 3-year local tumor progression than RFA, while maintaining equivalent 5-year overall survival [57]. Notably, CRA’s benefit in local control was most pronounced for larger tumors which is a scenario often accompanying PVTT, suggesting cryotherapy can effectively ablate substantial tumor masses with acceptable recurrence rates. This high-level evidence has helped validate CRA as a curative-intent therapy in HCC, lending confidence to its use in more advanced cases.

CRA in PVTT is still an emerging field with limited data. One study described a CRA technique that directly targets the tumor thrombus within the left medial portal vein of a patient with treatment-refractory HCC. The intravascular lesion (initially 22.8 × 18 mm) was ablated and at the one-month follow-up, showed a substantial reduction in thrombus size to 7.7 mm, demonstrating a cryoreductive response to CRA. Of note, the vessel patency was preserved, and the procedure was tolerated well with no major complications. This technique provides a foundation that CRA is a possible minimally invasive, liver sparing option for select patients with PVTT [58]. There are also formal trials underway evaluating CRA + camrelizumab combination specifically in HCC with PVTT (NCT04777802). CRA has re-emerged as a valuable modality for HCC with PVTT. While no randomized trial specifically in HCC with PVTT has been completed, ongoing studies are exploring CRA in these high-risk patients, and preliminary results are encouraging for patients who are candidates for local therapy but have tumors in locations less amenable to heat.

### Overview and Historical Context of Radiotherapy

Historically, standard algorithms recommended only systemic therapy, with locoregional treatments (TACE or TARE) playing a minor role [59, 60]. However, earlier eras of whole-liver irradiation caused severe liver toxicity (radiation-induced liver disease, RILD), and EBRT was largely abandoned. In recent decades, partial-liver techniques revived interest in RT for HCC where HBV-related HCC is prevalent [60, 61]. Notably, major Western guidelines still omit EBRT for PVTT, though evidence for SBRT is gradually emerging [60, 62].

**Fig. 6:**
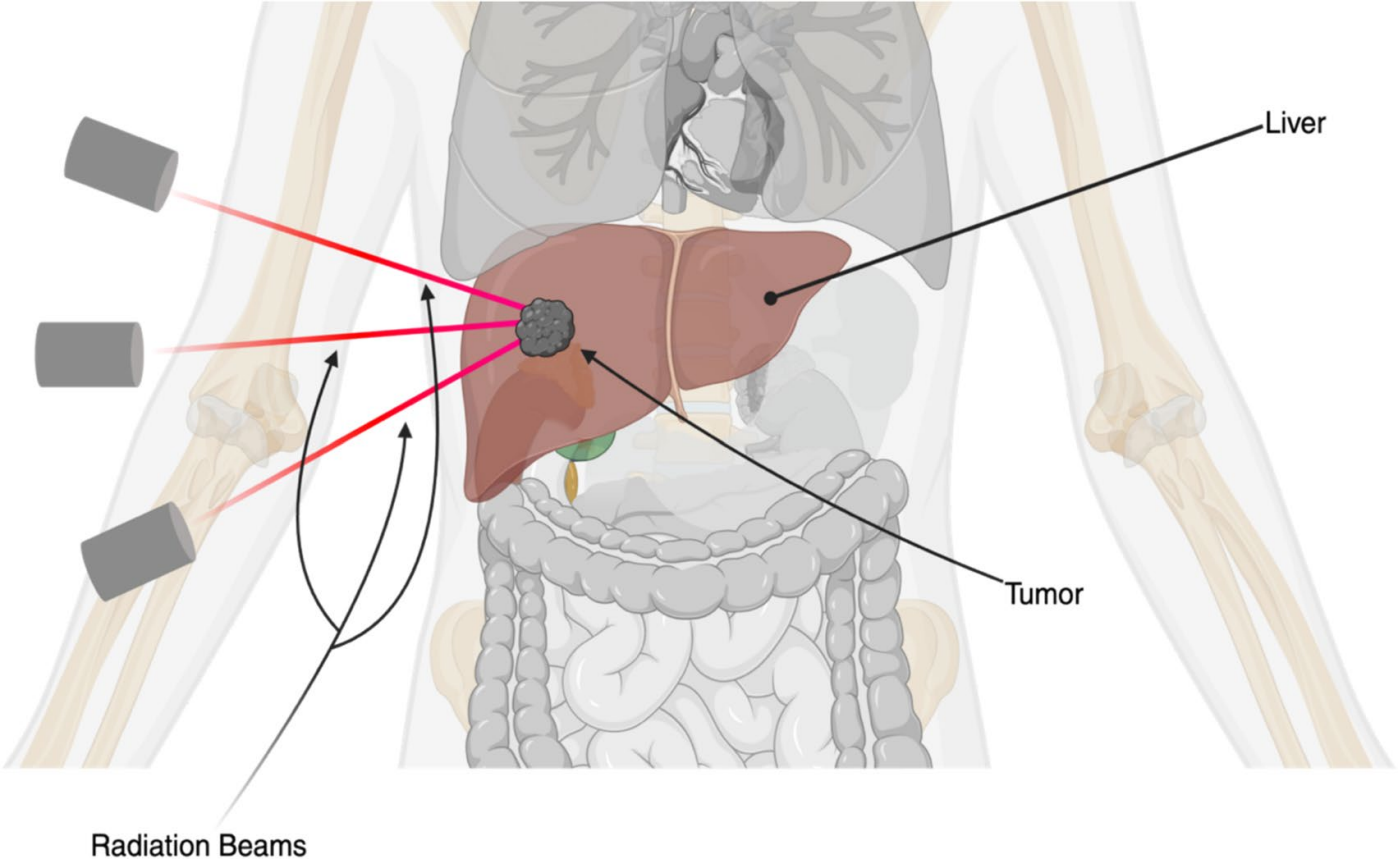
EBRT Targeting HCC. Multiple radiation beams converging to maximize the local dose while minimizing exposure to surrounding healthy tissue. Created in BioRender. Singh, R. (2025) https://BioRender.com/nw5qv5j

Advances in image guidance and motion management have been crucial. In a Japanese cohort of 116 patients with advanced PVTT, PBT (median ~ 72.6 Gy [RBE] in ~ 22–30 fractions) achieved 5-year local control 86.1% and median survival > 20 months (5-yr OS 25.1% in definitive cases) [62]. Overall, modern RT techniques deliver ablative dose to tumor while limiting liver dose, enabling EBRT to be applied safely in many patients as shown in Fig. 6.

### Radiotherapy in PVTT

Radiotherapy is often used in combination with other modalities and across trials, median OS frequently exceeds 10 months, with 1-year OS often in 40–60% of cases. In a large propensity-matched analysis (*n* = 610) from Korea, liver-directed combined RT (mostly 3D Conformal Radiation Therapy (3DCRT)/Intensity Modulated Radiation Therapy (IMRT), median dose 45–50 Gy) was compared to sorafenib alone. Median OS was 10.6 vs 4.2 months favoring RT (*p* < 0.001) [59]. This suggests EBRT substantially prolongs survival and downstages disease.

In several studies, RT + TKI was compared against TKI alone. In one Chinese series (*n* = 152), RT + Lenvatinib (L) was compared to RT + sorafenib (S). For Cheng’s Type I/II PVTT, median OS was 19.8 vs 13.5 months (L vs S, *p* = 0.047) and PFS 12.3 vs 7.3 months (*p* = 0.042). Even for Type III/IV PVTT, OS was 14.4 vs 8.3 months (*p* = 0.030) and PFS 8.3 vs 6.2 months (*p* = 0.026) [63]. These data indicate that RT + Lenvatinib may outperform RT + sorafenib, extending survival especially in lower PVTT grades. Another study analyzed Lenvatinib alone (*n* = 77) vs Lenvatinib + SBRT (*n* = 37). The combination yielded median OS 19.3 vs 11.2 months (*p* < 0.001), PFS 10.3 vs 5.3 months (*p* < 0.001), and ORR 56.8% vs 20.8% [64]. Subgroup analyses showed significant benefit in both Vp1–2 and Vp3–4 PVTT categories with manageable toxicity [64].

In summary, EBRT using 3DCRT, IMRT, SBRT, or protons has become an important tool against HCC with PVTT. Modern series report substantial improvements in survival and local control when EBRT is employed, especially as part of multimodality therapy [59, 65]. With careful patient selection and advanced delivery techniques, EBRT offers a potent option in treatment algorithms, often achieving tumor responses and symptom control with acceptable toxicity [60, 66].

## Future Directions by Locoregional Therapy for HCC with PVTT

### TACE: Next Generation Drug Delivery and Imaging Integration

Emerging trials are testing multimodal TACE combinations which show prolonged survival compared to TACE + Lenvatinib alone [67]. On the technical side, novel embolic material and imaging are rapidly maturing, and DEBs are increasingly used. Innovations include radiopaque microspheres and biodegradables that allow repeat treatment. Radiomics and machine learning models are also being developed to predict response and personalize dosage [68]. Next-generation TACE will exploit smarter delivery systems and image‐guided planning, likely improving efficacy in PVTT while limiting toxicity [68, 69].

### TAE: Refining Bland Embolization

Although chemoembolization dominates intermediate-stage HCC, there is renewed interest in bland TAE for PVTT. A 2024 meta‐analysis of RCTs found no significant difference in overall or PFS between TAE and TACE [70]. Patient selection will be key, and TAE may be favored for patients with poor tolerance to chemotherapy or contraindications to embolic drugs. New embolic particles such as imageable beads and calibrated microspheres that penetrate tumor microvasculature are under investigation [71]. In summary, TAE remains a viable approach, and combination of systemic therapy or sequential TACE, is expected to play a niche but important role in advanced HCC management [70].

### TARE: High-Dose Radiotherapy and Radioisotope Innovations

TARE is gaining traction for PVTT. Key developments focus on personalized dosimetry and immunotherapy combinations. Another frontier is Holmium-166 microspheres, which allow MRI-based dosimetry. A 2024 series reported that ^166Ho-TARE with personalized planning achieved a median OS of ~ 17 months and showed a dose–response correlation [72]. Holmium’s paramagnetic properties may further refine dose delivery and follow-up imaging. Next-generation TARE for PVTT will feature high-precision, boosted-dose treatments, new isotopes like ^166Ho, and rational combination with systemic agents. These innovations promise better local control and potentially longer survival in segmental/lobar PVTT [36, 62, 73].

### HAIC: Optimized Intra-arterial Chemotherapy

HAIC has long been used in East Asia and continues to evolve for PVTT. Recent practice has found that adding TKIs and PD-1 inhibitors to HAIC markedly increased tumor response and survival in PVTT patients [74]. New prospective trials are underway (HAIC + Lenvatinib + anti-PD1) to confirm these combinations. Novel port-catheter systems and microcatheters allow more uniform drug distribution and potentially outpatient therapy. Ongoing work aims to optimize infusion rates and schedules guided by computational flow models.

Expert consensus is coalescing around combining HAIC with systemic therapy for PVTT. Going forward, the emphasis will be on well‐designed trials and biomarker-driven patient selection. In summary, HAIC is likely to remain a mainstay loco-regional option for PVTT, with advances in catheter technology, drug formulations, and combined modality protocols enhancing its effectiveness [75].

### Precision Radiotherapy and Immune Synergy

EBRT is a precision tool for PVTT and new hypo-fractionated regimens and particle therapies offer powerful options [61]. These results are prompting broader use of SBRT/PBT in guidelines. AI‐based auto-planning and organ-at-risk segmentation accelerate treatment planning and allow daily plan adaptation. Moreover, combinations of RT with novel agents are a hot area, and immunologic effects of ablative RT on PVTT may be harnessed by checkpoint inhibitors or anti-angiogenics. A forthcoming systematic review reports that EBRT + Immune checkpoint inhibitor (ICI) yields ORRs > 50% and DCRs ~ 88% [76]. In summary, radiotherapy for HCC with PVTT is expected to become more customized and integrated. Ultra‐conformal SBRT and PBT will be delivered with image‐guided precision, potentially even in fractionated boost schedules for bulky PVTT. Modern RT may shift treatment of segmental PVTT toward local control, complementing or even deferring systemic therapy [61, 62].

### Ablation: Advanced Image-Guided Techniques

Thermal and non-thermal ablation are expanding their role. For PVTT specifically, only very focal branches can be safely ablated, nonetheless, innovations will increase precision for vascular involvement. Irreversible electroporation (IRE) (NanoKnife) is one such advance demonstrating its noninferiority to RFA for tumor control in liver tumors, and IRE’s non-thermal mechanism may allow safer ablation near vessels [77]. Image guidance is a key frontier. Robotic needle-guidance platforms (EPIONE) and CT/MRI fusion are now available for liver ablation, enabling more accurate targeting of complex lesions [78]. Radiomics and AI may further refine ablative zone prediction, helping operators ensure complete coverage with minimal overlap. While ablation for PVTT is niche, these advances improve its feasibility and safety. We expect that robotic and image‐guided ablation will become more common for selected PVTT cases or for intraportal metastases.

### Nanotherapy: Targeted Nanoparticles and Theranostics

Nanoparticle (NP) formulations are especially attractive in HCC with PVTT because carriers can penetrate tumor microenvironments and sustain the release of chemotherapeutics at the PVTT site, potentially overcoming the limitations of free drugs [75, 79]. There are several preclinical studies that analyze this therapy and have demonstrated a dramatic tumor regression, leading to complete disappearance of lesions in 2/3 rats by 14 days [75]. Similarly, another study emulsified lipiodol with a pH-sensitive hexahistidine–zinc metal–organic nanocarrier (HmA) loaded with DOX (DOX@HmA/Lipiodol) for TACE [80]. The results showed in rabbits markedly suppressed metastasis and lung metastases were nearly absent in the HmA-NP group whereas the DOX/lipiodol and saline groups had numerous nodules [80]. Other platforms also achieved promising technological advancements with one study achieving > 50% transfection of orthotopic HCC tumors with negligible uptake in normal liver [81]. Each of these platforms exemplifies innovations in carrier design tailored to locoregional delivery in HCC, highlighting the potential of local nanotherapy even in the challenging PVTT context.

Advancements in nanotechnology are slowly paving the way for targeted therapy in HCC by offering low toxicity, biodegradability, and biocompatibility of nanomaterials. Challenges include manufacturing complexity, regulatory hurdles, and cost. Additionally, the complex micro-nano delivery system requires further significant optimization to develop efficient carriers for radiodiagnosis and treatment of HCC [82]. Finally, PVTT itself adds complexity and to date, few studies have explicitly addressed PVTT targeting beyond the concept of delivering anticoagulant + chemotherapeutic. Thus, while the translational potential is high, achieving such a feat will demand rigorous optimization of NP design, thorough safety evaluation, and ultimately clinical trials to assess benefits in PVTT patients.

## Conclusion

Each locoregional modality for HCC with PVTT is being re-envisioned. HCC surveillance should focus on advancing models that integrate clinical, genetic, and molecular factors that better stratify risk and tailor surveillance strategies [83]. This review proposes a stratified treatment approach for HCC with PVTT based on PVTT classification and liver function, integrating current evidence for locoregional and combination therapies. The proposed framework may guide treatment selection, and support decision-making for advanced HCC management. Future clinical research must confirm the optimal combinations and sequencing of locoregional modalities, especially in the era of immunotherapy. Innovation should continue with developing novel drug‐delivery (nanoparticle) strategies, personalizing dosimetry, and refining PVTT risk stratification to identify who benefits most. Sustained investment in clinical trials and technological advances will be essential to fully realize and integrate these treatments into a modern, multidisciplinary care paradigm [84, 85].

## Data Availability

No datasets were generated or analysed during the current study.

## Abbreviations

*3DCRT*: 3D conformal radiation therapy
*BCLC*: Barcelona Clinic Liver Cancer
*CP*: Child-Pugh
*CRA*: Cryoablation
*DEB*: Drug-eluting bead
*EBRT*: External beam radiation therapy
*HAIC*: Hepatic arterial infusion chemotherapy
*HCC*: Hepatocellular carcinoma
*ICI*: Immune checkpoint inhibitor
*IMRT*: Intensity-modulated radiation therapy
*MRI*: Magnetic resonance imaging
*MWA*: Microwave ablation
*ORR*: Objective response rate
*OS*: Overall survival
*PBT*: Proton beam therapy
*PD-1*: Programmed Death-1
*PFS*: Progression-free survival
*PS*: Performance status
*PV*: Portal vein
*PVTT*: Portal vein tumor thrombus
*PVR*: Portal vein recanalization
*RCT*: Randomized control trial
*RFA*: Radiofrequency ablation
*RT*: Radiotherapy
*SBRT*: Stereotactic body radiation therapy
*TACE*: Transarterial chemoembolization
*TAE*: Transarterial embolization
*TARE*: Transarterial radioembolization
*TKIs*: Tyrosine kinase inhibitors
*US*: Ultrasound
^90^*Y*: Yttrium-90
